# Chemical Characterization, Bioactivity and Toxicity of European Flora Plant Extracts in Search for Potential Natural Origin Preservatives

**DOI:** 10.3390/plants12152784

**Published:** 2023-07-27

**Authors:** Breno Martins de Deus, Conceição Fernandes, Adriana K. Molina, Virginie Xavier, Tânia C. S. P. Pires, Filipa Mandim, Sandrina A. Heleno, Tiane C. Finimundy, Lillian Barros

**Affiliations:** 1 Centro de Investigação de Montanha (CIMO), Instituto Politécnico de Bragança, Campus de Santa Apolónia, 5300-253 Bragança, Portugal; brenodeus@alunos.utfpr.edu.br (B.M.d.D.); conceicao.fernandes@ipb.pt (C.F.); amolina@ipb.pt (A.K.M.); virginie.xavier@ipb.pt (V.X.); tania.pires@ipb.pt (T.C.S.P.P.); filipamandim@ipb.pt (F.M.); sheleno@ipb.pt (S.A.H.); lillian@ipb.pt (L.B.); 2 Laboratório Associado para a Sustentabilidade e Tecnologia em Regiões de Montanha (SusTEC), Instituto Politécnico de Bragança, Campus de Santa Apolónia, 5300-253 Bragança, Portugal; 3 Nutrition and Bromatology Group, Department of Analytical Chemistry and Food Science, Faculty of Science, Universidad de Vigo, E32004 Ourense, Spain

**Keywords:** phenolic compounds, plant extracts, natural preservatives, bioactivity, toxicity, *Artemia franciscana*, European flora

## Abstract

Consumer demand for natural and healthier products has led to an increasing interest in the bioactive and therapeutic properties of plant extracts. In this study, we evaluated the phenolic compounds profile, bioactivities, and toxicities of plant extracts from eight European flora species, including *Calendula officinalis* L., *Calluna vulgaris* (L.) Hull, *Hippophae rhamnoides* L., *Juglans regia* L., *Mentha cervina* L., *Rubus idaeus* L., *Sambucus nigra* L., and *Vitis vinifera* L. The aim was to identify potential preservatives of natural origin. Phenolic compounds were identified by HPLC-DAD-ESI-MS. Caffeic acid derivatives, ellagitannins, flavonols, and flavones were the major phenolic compounds identified. The total phenolic content varied from 16.0 ± 0.2 (*V. vinifera*) to 123 ± 2 mg/g (*H. rhamnoides*) of dry extract. All extracts showed antioxidant potential and exhibited activity against some of the microorganisms tested. *S. nigra* showed the highest activity in the inhibition of oxidative hemolysis (OxHLIA) assay and *H. rhamnoides*, notably, had the lowest IC_50_ values in TBARS and DPPH assays, as well as the lowest minimum inhibitory concentration (MIC) values. Regarding in vitro cytotoxicity, in tumor and non-tumor cell lines, although some extracts revealed toxicity against normal cells, it was found that the samples *C. vulgaris*, *V. vinifera* and *R. idaeus* might be used against tumor cells since the active concentration is much lower than the one causing toxicity. In vivo acute toxicity tests using *Artemia franciscana* suggest low toxicity for most extracts, with LC_50_ > 400 mg/L. These results showed the potential of the studied extracts as natural preservatives, given their richness in compounds with bioactive properties, highlight their potential value to the production chain.

## 1. Introduction

Prominent among phytochemicals are phenolic compounds, an abundant group of compounds that are biosynthesized by plants in response to environmental stresses and for metabolic functions, being directly associated with bioactive properties such as antioxidant, anti-inflammatory, antitumor and antimicrobial activities [1,2,3]. As the world grapples with mounting health concerns and an increasing focus on environmental sustainability, the search for safer and more sustainable preservatives has become a paramount endeavor for industries worldwide. Phenolic compounds present an appealing solution to this challenge, not only due to their bioactive properties but also because of their potential as natural and environmentally friendly alternatives for preservation. In the extraction of phenolic compounds involving solvents, the polar ones are considered more efficient, in which ethanolic and hydroethanolic solutions are more sustainable (“green” solvents from renewable sources), allowing the preservation of the quality of the final extract [1,2,3,4,5]. Plant extracts, rich in phenolic compounds, have been recognized for their diverse bioactive properties, including antioxidant, anti-inflammatory, antitumor, and antimicrobial activities, making them highly desirable for use in food, cosmetic, and therapeutic applications [6,7,8].

Incorporating the natural power of European flora into everyday products can make them safer for health and the environment. In this pursuit, we delve into the diverse world of plant extracts, uncovering their bioactive secrets, assessing their safety, and discovering their potential as greener preservatives [9,10,11,12,13,14,15,16].

Biological processes (such as cellular respiration) and external factors (such as stress, xenobiotic exposition, and radiation incidence) can lead to the formation of Reactive Oxygen Species (ROS) in the body. Such species cause structural damage to the cells and are understood as precursors to the development of inflammatory processes, cancer, and the aging process [1,12,15,17,18,19,20]. Phenolic compounds present antioxidant activity, acting by neutralizing free radicals through different mechanisms, thus protecting the organism [21]. In the food industry, antioxidants are employed with the purpose of increasing the shelf life of products, especially those containing unsaturated lipids in their composition, which are more susceptible to the oxidative action of free radicals.

The presence of fungi and bacteria, which can cause food-borne infections and intoxications to the consumer, is a constant concern in industry, compromising products’ quality and safety. Furthermore, contaminations cause processes that lead to changes in the characteristics of the products, generating waste and financial losses [3,8,13]. Phenolic compounds may present antimicrobial activity (fungistatic, fungicidal, bacteriostatic, and bactericidal), making them interesting agents for food and cosmetic industries to replace artificial preservatives. Plant extracts have long been revered for their medicinal properties, and their use as natural additives in the food and cosmetic industries is gaining popularity. Consumers now demand safer products without compromising on quality, and this has fueled the search for natural-origin preservatives. Moreover, mounting concerns about the environmental impact of synthetic additives drive the need for greener alternatives [11,17,22].

Our research not only contributes to the understanding of the bioactivity and safety of plant extracts, but also offers potential solutions for industries seeking greener alternatives. Moreover, our study expands the knowledge base on European flora, which could have broader implications for conservation efforts and sustainable utilization. In vitro tests are important for providing an initial overview of organisms’ response to the substance, and are widely used to study the therapeutic potential of compounds of biological or synthetic origins [22]. In vivo assays allow a more complete and systemic evaluation of the toxicological effects of a substance in a test organism, representing important steps in validating the safety of using a given substance for different purposes [23,24]. The brine shrimp *Artemia franciscana* (formerly *Artemia salina*) has been widely used for the determination of the in vivo acute toxicity of substances due to the ease of obtaining viable individuals at any time of the year from dormant eggs (cysts) that can be stored for a long time, among other advantages [25]. The majority of toxicity tests are conducted in vitro due to their widespread use, cost-effectiveness, speed, and simplicity. However, in vivo assays offer a more comprehensive understanding of the overall response of a living organism to one or a set of substances, including potential synergistic effects between components. Building upon existing research on the bioactivity and properties of plant extracts, this study expands the scope by incorporating both in vitro and in vivo toxicity assessments, providing a comprehensive evaluation of their safety and potential. Our primary objectives are to chemically characterize various European flora extracts, evaluate their bioactive properties, assess their toxicity through in vitro and in vivo assays, and explore their potential as natural preservatives. In doing so, we aim to contribute to the search for safer and sustainable alternatives to synthetic additives. The plants selected in this study are native to Europe, namely, *Calendula officinalis* L., *Calluna vulgaris* (L.) Hull, *Hippophae rhamnoides* L., *Juglans regia* L., *Mentha cervina* L., *Rubus idaeus* L., *Sambucus nigra* L. and *Vitis vinifera* L. By adopting a holistic approach to these plant extracts native to Europe, this study adds a novel dimension to the exploration of natural preservatives, with the potential to revolutionize industries seeking safer alternatives.

## 2. Results and Discussion

### 2.1. Profiles in Phenolic Compounds

Results regarding the identification and quantification of the phenolic compound profiles in the plant extracts are presented in Table 1. It was possible to tentatively identify 114 compounds, including phenolic acids (highlight to caffeoylquinic acids), flavan-3-ols, (+)-catechin, (*epi*)catechin dimers, ellagitannins and flavonoid glycosides, which have been extensively described previously in relation to the studied plant species [1,6,26,27,28,29,30,31,32,33,34,35,36,37,38].

**Figure 1:**
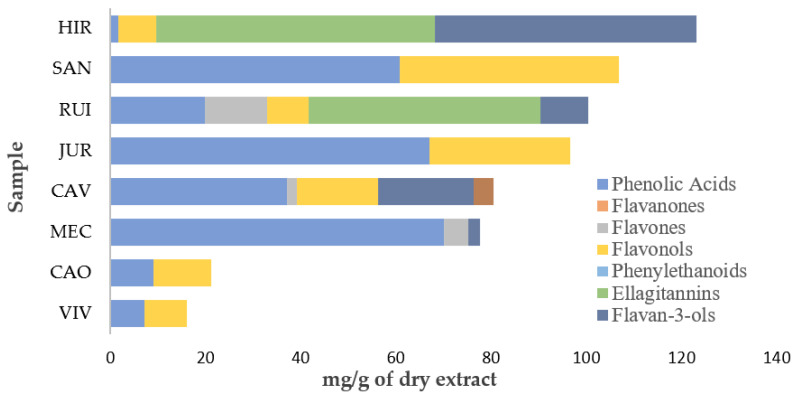
Quantification of the classes of phenolic compounds identified in the extracts. CAO, *C. officinalis*; CAV, *C. vulgaris*; HIR, *H. rhamnoides*; JUR, *J. regia*; MEC, *M. cervina*; RUI, *R. idaeus*; SAN, *S. nigra*; VIV, *V. vinifera*.

When evaluating the quantification of total phenolic compounds, the highest concentrations obtained were for HIR (123 ± 2 mg/g extract), SAN (106 ± 2 mg/g extract) and RUI (100 ± 2 mg/g extract), as observed in Figure 1.

When analyzing the potential of the studied species to be configured as sources of phenolic compounds of a particular class, the high contents of phenolic acids in MEC, JUR and SAN samples stand out. Ellagitannins were the major compounds in HIR, corresponding to approximately 48% of quantified phenolic compounds. Suvanto et al. (2018) [39] studied ellagitannin composition in *H. rhamnoides* leaf extracts using a 70% water/acetone solution (*v*/*v*). The findings presented in this work regarding ellagitannins concentrations are consistent with the range presented by these authors (between 42.5 and 109.1 mg/g of dry extract).

It is important to note that for the RUI extract, the presence of a few compounds, namely, lambertianin C and galloyl-bis-HHDP-glucoside, in high amounts led ellagitannins to be the most representative class, while a greater diversity of components quantified in lower proportions made this plant profile diverse in phenolic acids and flavonols. A similar situation is observed for CAV, in which the abundance of the dimer of 3-*O*-caffeoylquinic acid led the phenolic acids to be the most representative class, whereas a greater diversity of compounds is seen in flavonols.

Other works have already studied the profile of phenolic compounds of *C. vulgaris*. The work performed by Starchenko et al. (2020) [6], which evaluated the aqueous and hydroethanolic extracts of the aerial parts of *C. vulgaris* obtained from extraction with temperatures between 80 and 90 °C, reported a higher content of hydroxycinnamic acid derivatives, such as chlorogenic and caffeic acid derivatives, which agrees with our results. The work of Mandim et al. (2018) [32] evaluated extracts from the extremities of *C. vulgaris* flowers obtained from maceration extractions making use of different organic and aqueous solvents. The profile of compounds obtained by these authors shows a higher content of flavonoids, obtaining a distinct profile from the one obtained in this work.

For *C. officinalis*, the work of Miguel et al. (2016) [34] evaluated the profile of the aqueous (obtained by infusion) and hydromethanolic (obtained by extraction under magnetic stirring at room temperature) extracts of the flowers. The authors reported a predominance of flavonols content, which is in line with that found in this work. Most of the identified compounds here were also identified by these authors, except for quercetin-7-*O*-malonyl-hexoside and kaempferol-*O*-rutinoside. In this same work by Miguel et al. (2016) [34], the extracts of *M. cervina* leaves (obtained through the same extraction techniques mentioned before) were also characterized. The authors found very expressive amounts of phenolic acids (10.53 ± 0.13 mg/g extract), which agrees with the findings in the present work; however, they did not identify other classes of phenolic compounds, such as flavones and flavan-3-ols.

The work of Zheng et al. (2019) [31] presented results of the characterization of hydromethanolic extracts from various parts of *H. rhamnoides* (including branches, leaves, and fruits) obtained by ultrasonic extraction. The results indicate that the content profile of the branches and leaves show greater similarity, whereas the fruits composition has shown greater differences. The profile obtained by these authors differs from that obtained in this work. 

Regarding *J. regia*, the work of Amaral et al. (2004) [30] with hydroethanolic extracts of the leaves also reported the highest content of phenolic acids. The authors obtained a different profile from the one identified in this work.

The work of Pavlović et al. (2016) [40] made an evaluation of the phenolic compound profile of the hydromethanolic extracts of the leaves of *R. idaeus*, obtained in ultrasonic extraction and at room temperature. Most of the compounds identified in the present work were previously identified by these authors. 

In another study performed by Skowrońska et al. (2022) [26], the authors evaluated the phenolic compound profiles of hydroethanolic extracts of *S. nigra* leaves obtained from cold (maceration) and hot (decoction) extractions. The results were in agreement with the ones described in the present work regarding the content of phenolic acids, with the leaves being the plant parts with the highest amounts of these components. Dawidowicz et al. (2006) [20] evaluated the hydroethanolic extracts of the leaves, flowers and fruits of *S. nigra* obtained from pressurized liquid extraction, with emphasis on the detection of flavonols. The results obtained by these authors suggest the presence of a significant content of rutin derivatives (flavonols) in leaves, which was also observed in the present work. 

The leaves of *V. vinifera* were also the subject of study in the work of Fernandes et al. (2013) [35], the extraction being performed by decoction. The profile of phenolic compounds obtained in this work was distinct from that obtained by the cited authors. 

Generally, our findings regarding the phenolic compounds profile align with previous studies on the studied plant species [1,6,26,27,28,29,30,31,32,33,34,35,36,37,38], which have reported the presence of phenolic acids, flavan-3-ols, ellagitannins, and flavonoid glycosides. The variations observed in the composition of phenolic compounds could be attributed to factors such as plant part, extraction technique, and geographic origin, as also reported in other studies [6,32,34,40].

### 2.2. Bioactive Properties

The results for antioxidant activity are presented in Table 2 in terms of the concentration leading to 50% inhibition of the oxidative mechanism (IC_50_; µg/mL).

From the data presented in Table 2, it is possible to observe that all extracts presented antioxidant activity under all methods, with higher activity when lower concentrations were needed to inhibit 50% of oxidation.

Compared to the IC_50_ value of 23 ± 0.4 µg/mL for Trolox in the TBARS assay, the extracts of some plants showed similar or even better performances, such as extracts VIV, CAV and RUI; HIR showed a performance approximately 19.1 times better than Trolox. The high antioxidant potential of the HIR extract has been previously evaluated by the TBARS method, and has applications in the preservation of products with high lipid content, as in the work of Salejda et al. (2014) [41] on the improvement of pork meat preservation. In the DPPH assay, all samples (except CAO) presented better performances than Trolox. For the OxHLIA assay, considering a Δt of 60 min, CAV and VIV extracts showed similar results compared to Trolox. The SAN extract was able to delay oxidative hemolysis for 120 min with results comparable to Trolox.

The results obtained for CAV are very similar to those reported in the literature, for example in the work of Rieger et al. (2008) [38], where a range between 8.2 and 9.4 µg/mL (for the hydroethanolic extract) was obtained in the DPPH assay, and in the work of Cucu et al. (2022) [2], who obtained the value of 8 ± 0.2 µg/mL in the TBARS assay (for the acetone extract of the inflorescences) and 34.0 ± 0.6 µg/mL in the OxHLIA assay at Δt = 60 min (for the methanolic extract of the inflorescences). 

Finally, comparing our antioxidant activity results with other studies, we found that several plant extracts exhibited remarkable antioxidant potentials, surpassing the activity of the synthetic antioxidant Trolox [41,42,43]. The high content of phenolic acids in certain samples, such as MEC, JUR, and SAN, could be linked to their superior antioxidant performance [38]. Additionally, our findings are consistent with previous research on the antioxidant activity of *S. nigra, V. vinifera*, and *C. vulgaris* extracts, further reinforcing the potential to use these plant species as valuable sources of natural antioxidants [2,26,35].

The results for antimicrobial activity are presented in Table 3 and expressed in terms of the minimum inhibitory concentrations (MIC; mg/mL) at which bacteriostatic/fungistatic activity was observed. No bactericidal (MBC) or fungicidal (MFC) effect was detected in the tested extracts.

From the results presented in Table 3, it is possible to observe that only HIR and RUI presented antimicrobial activity against all the tested microorganisms. The work of Othman et al. (2021) [44] evaluated the antimicrobial activity of quince (*Cydonia oblonga* Mill.) peel extracts on a similar group of representatives of food-contaminating bacteria in comparison with the synthetic additives sodium benzoate (E211) and potassium metabisulfite (E224), employed as preservatives in the food and cosmetic industries. Considering the MIC values obtained by these authors for the additives, in the range of 4.0–0.5 mg/mL for E211 and 2–0.5 mg/mL for E224, it is possible to infer that the results obtained for HIR extract (Table 2) represent a high potential for substituting synthetic preservatives. This potential is also reinforced by the response observed for the microorganisms *S. aureus* and *L. monocytogenes* (MIC = 0.6 mg/mL), which were comparable to or even better than the activities of the artificial additives (E211: MIC = 4.0–1.0 mg/mL; E224: MIC = 1.0–0.5 mg/mL). 

*E. coli* and *P. aeruginosa* were the bacterial strains that showed the highest resistance, which was also observed in the work of Baydar et al. (2004) [45] in the evaluation of antimicrobial activity for *V. vinifera* extracts. In general, it can be observed that Gram-positive bacteria were more affected, since bacteriostatic effects were exhibited by all the extracts. This finding is in line with what was also observed by Cucu et al. (2022) [2] when evaluating the antimicrobial activity of *C. vulgaris* extracts on different bacteria. Gram-negative bacteria are inherently more resistant to external agents due to the presence of lipopolysaccharides in their outer membranes, while the peptidoglycan layer present in Gram-positive bacteria is not configured as such an effective permeability barrier [42]. The antimicrobial activity against various microorganisms observed in our results highlights the potential of HIR and RUI extracts as promising natural alternatives to synthetic preservatives, as their MIC values were comparable, or even superior, to those of commonly used synthetic additives [44]. The differential response of Gram-positive and Gram-negative bacteria to our extracts aligns with similar observations reported in other studies [2,45].

Regarding fungi, all extracts showed fungistatic activity, highlighting the activity of the JUR extract against *A. brasiliensis*. The high antifungal potential of *J. regia* against this fungus has been previously reported in the work of Bennacer et al. (2022) [43], in which the tannic extract of the leaves was tested using the disk-diffusion method. Furthermore, the antifungal potential of JUR against *A. brasiliensis*, as observed in our study, is consistent with findings from a previous study focusing on the same plant extract [43].

### 2.3. In Vitro and In Vivo Toxicity

The results regarding cytotoxic activity are presented in terms of the concentration leading to 50% inhibition of cell proliferation (GI_50_; µg/mL) in Table 4, as well as results of acute toxicity in *Artemia franciscana*, expressed as the range in which the value of the concentration lethal to 50% of the population of individuals (LC_50_; mg/L) in 24 h is found.

#### 2.3.1. Cytotoxicity

From the results presented in Table 4, for non-tumor cell lines, we can see that higher GI_50_ values are preferable, suggesting greater safety for the integrity of normal cells, while lower GI_50_ values represent higher cytotoxic activity, this result being preferable for tumor cell lines.

In establishing a comparison between the response of normal lines and tumor lines for the same extract, it is possible to highlight the inhibitory effect that VIV, CAV and RUI presented on the AGS line, verifying the inhibition of tumor cell proliferation at concentrations considerably lower than those configured as inhibitory to normal lines. For the other extracts, the GI_50_ values for at least one of the normal cell lines turned out to be lower than what is necessary to cause inhibitory effects on tumor cells.

While the present results suggest cytotoxic effects on renal cells for JUR (VERO; GI_50_ = 69 ± 1 µg/mL), for SAN and VIV extracts, higher GI_50_ values were obtained. The extract of the SAN sample showed no activity for any of the tumor lines at the concentrations tested. In the work of Noumi et al. (2010) [46], the bioactivities profile of *S. nigra* fruit juice was evaluated, with an emphasis on anthocyanins, where it was found to be more innocuous to PLP_2_ cells (GI_50_ > 400 µg/mL) and to show high cytotoxic responsiveness to tumor cells, for example cervical carcinoma, lung carcinoma and breast adenocarcinoma cells (HeLa, NCI-H460 and MCF-7 lines, respectively), with GI_50_ values in the range between 16 ± 1 and 58 ± 1 µg/mL. These findings differ from those found in the present work, which can be explained by the use of different parts of the plant. The VIV extract showed no activity for CaCo-2 and MCF-7 lines, and no activity for any of the normal cell lines, which is in agreement with the findings of Colombo et al. (2019) [47] in their study on the bioactive properties of grape pomace.

#### 2.3.2. In Vivo Acute Toxicity

No greater than 10% mortality in individuals was observed in the positive control of the tests. The negative control used (K_2_Cr_2_O_7_) attests to the sensitivity of the batch of test organisms, and the calculated value of LC_50_ (48 ± 4 mg/L) is consistent with mortality as a function of increasing concentration. Tapia-Salazar et al. (2022) [48] reported LC_50_ values in the range between 9 and 78 mg/L for *A. salina*. 

Using the classification criteria according to Meyer et. al. (1982) [49], CAV and CAO extracts showed moderate toxicity (Table 4), while the other extracts showed probable low toxicity. Differences between in vitro and in vivo assays can be justified by the toxicological effect on the whole organism (*A. franciscana*) compared to on the cell. The moderate toxicity observed for CAV and CAO extracts in our cytotoxicity assays is in accordance with previous studies, which also reported cytotoxic effects on certain cell lines [2,46,50]. Conversely, the low toxicity observed in SAN and VIV extracts is congruent with their favorable in vivo toxicity response, suggesting a potentially safer profile. Our in vivo results are supported by other investigations indicating that compounds with antitumor potential or pesticide applications may exhibit toxic effects on *Artemia franciscana*. These results emphasize the importance of considering both in vitro and in vivo toxicity assessments to comprehensively evaluate the safety profile of the plant extracts.

## 3. Materials and Methods

### 3.1. Plant Material

Samples of *Calendula officinalis* L. flowers (CAO), *Juglans regia* L. leaves (JUR), *Mentha cervina* L. leaves (MEC), *Rubus idaeus* L. leaves (RUI), *Sambucus nigra* L. leaves (SAN) and *Vitis vinifera* L. leaves (VIV) were obtained from ERVITAL—Plantas Aromáticas e Medicinais, Viseu, Portugal. *Calluna vulgaris* (L.) Hull stems, leaves, and flowers (CAV), and *Hippophae rhamnoides* L. branches, twigs, leaves and flowers (HIR), were obtained from Welzow, Germany. All samples were dried at 40 ± 0.5 °C for 48 h and subjected to grinding.

### 3.2. Hydroethanolic Extracts

For each gram of sample weighed, 30 mL of ethanol/water mixture in the proportion 80:20 (*v*/*v*) was added as solvent. The extraction was carried out under magnetic stirring and at room temperature for 1 h, filtered (Whatman n° 4), reserving the liquid portion in a flask and returning the solid part to the beaker, and then repeating the process, according to Yoo et al. (2022) [51]. Afterwards, the solid portion was discarded, and the liquid portion was subjected to rotary evaporation (Büchi R-210, Flawil, Switzerland) under vacuum at a temperature not exceeding 40 °C until ethanol was removed. The remaining aqueous portion was frozen and subjected to freeze-drying (Freeze Dryer Telstar LyoQuest-55, Milan, Italy) for 48 h at −55 ± 0.5 °C.

### 3.3. Chemical Characterization

A known mass of 10 mg of extract was weighed and 2 mL of the ethanol/water mixture (80:20; *v*/*v*) was added. The solution was filtered with a 0.2 μm nylon syringe filter into a vial and analyzed on a Dionex Ultimate 3000 UPLC (Thermo Scientific, San Jose, CA, USA) system equipped with an automatic injector with temperature controlled at 5 °C, a degasser and a column compartment with the thermostat held at 35 °C, as performed by Alves-Ferreira et al. (2019) [52]. The compound detection was performed using a Diode Array Detector (DAD) at the wavelengths of 280, 330 and 370 nm. The compound separation was performed using a Waters Spherisorb S3 ODS-2 C18 column (4.6 × 150 mm; 3 μm; Milford, Massachusetts, USA) in reverse phase employing (A) formic acid (0.1%) and (B) acetonitrile in the mobile phase at a flow rate of 0.5 mL/min in the following gradient elution regime: 10 to 15% B for 5 min, 15 to 20% B for 5 min, 20 to 25% B for 10 min, 25 to 35% B for 10 min, 35 to 50% B for 10 min, and rebalancing the column for 10 min. The system was connected to an Ion Trap Linear LTQ XL mass spectrometer (Thermo Finnigan, San Jose, CA, USA) with an electrospray ionization (ESI) source employing nitrogen as the carrier gas at 50 psi, with a spray voltage of 5 kV, initial temperature of 325 °C, capillary voltage of −20 V and tube lens voltage of −66 V. Spectra were recorded in the negative mode between 100 and 1500 of charge mass ratio (*m*/*z*), and the collision energy was kept at 35 arbitrary units. Data acquisition, processing and interpretation were performed with Xcalibur software version 2.2 (Thermo Finnigan, San Jose, CA, USA). For identification of the compounds, retention time (Rt), wavelength of maximum absorption (λmax), pseudomolecular ion ([M-H]^−^), UV-Vis spectra, mass spectra and patterns of the ion breakdown (MS^2^) were compared with commercially available standards and those in the literature. For the components’ quantification (expressed in mg/g dry extract), calibration curves (R^2^ ≥ 0.999) obtained from available standards were used: caffeic acid (*y* = 388,345*x* + 406,369); rosmarinic acid (*y* = 191,291*x* – 652,903); apigenin-7-*O*-glucoside (*y* = 10,683*x* – 45,794); syringic acid (*y* = 376,056*x* + 141,329); apigenin-6-*C*-glucoside (*y* = 107,025*x* + 61,531); quercetin-3-*O*-glucoside (*y* = 34,843*x* – 160,173); chlorogenic acid (*y* = 168,823*x* – 161,172); ellagic acid (*y* = 26,719*x* – 317,255); gallic acid (*y* = 131,538*x* + 292,163); quercetin-3-*O*-rutinoside (*y* = 13,343*x* + 76,751); hydroxybenzoic acid (*y* = 208,604*x* + 173,056); catechin (*y* = 84,950*x* – 23,200); luteolin-6-*C*-glucoside (*y* = 4087.1*x* + 72589); *p*-coumaric acid (*y* = 301,950*x* + 6966.7); taxifolin (*y* = 203,766*x* – 208,383); (*epi*)catechin (*y* = 10,314*x* + 147,331) and ferulic acid (*y* = 633,126*x* − 185,462).

### 3.4. Antioxidant Activity

#### 3.4.1. Inhibition of Lipid Peroxidation by Thiobarbituric Acid Reactive Species (TBARS)

A given mass of pig brain (*Sus scrofa*) was weighed into a falcon tube and twice this mass of Tris-HCl buffer (20 mM; pH = 7.4) was added. After shaking, the tube was treated in the centrifuge (Centurion K24OR-2003 refrigerated, Centurion Scientific, Stoughton, Chichester, UK) at 3500 rpm for 10 min. As performed by Pinela et al. (2012) [53], in 48-well microplates, 200 µL of extract solution in the hydroethanolic mixture used in the extraction was added, and serial dilutions were performed in order to obtain 8 distinct concentrations, depending on the tested extract, in triplicate. The extraction solvent was used as the negative control and Trolox (a substance recognized for its antioxidant activity) was used as the positive control. In this sequence, 100 µL of ascorbic acid (0.1 mM), 100 µL of iron sulfate (10 mM), and 100 µL of the pig brain suspension supernatant were added to the wells. The plate was incubated at 37 ± 0.5 °C for 1 h. Afterwards, 500 µL of freshly prepared trichloroacetic acid (28%; *w*/*v*) and 380 µL of thiobarbituric acid (2%; *w*/*v*) were added. The plate was incubated at 80 ± 0.5 °C for 20 min. The content of each well was transferred to Eppendorf tubes that were centrifuged (Microfuge 16, Beckman Coulter, Brea, CA, USA) at 3000 rpm for 5 min. The supernatant was transferred to a 96-well plate and taken for absorbance reading in the spectrophotometer (SPECTROstar Nano, BMG LABTECH, Ortenberg, Germany) at a 532 nm wavelength. From Equation (1), the percentage of lipid peroxidation inhibition (I) can be determined.

I(%) = (A − B)/A × 100(1)

where A refers to the absorbance presented by the negative control and B to that presented by the extract. The results are expressed in terms of the concentration of extract able to inhibit lipid peroxidation by 50% (IC_50_), obtained from the correlation between the concentrations and the percentage of inhibition.

#### 3.4.2. Inhibition of Oxidative Hemolysis (OxHLIA)

In this assay, the inhibition of free radical-induced hemolysis was determined in a suspension of healthy sheep erythrocytes. The extract was dissolved in phosphate-buffered saline (PBS; pH = 7.4) and added (400 μL) into a 48-well plate, where serial dilutions were performed, obtaining 6 different concentrations ranging from 800 to 6.25 µg/mL, which were tested in triplicate. Distilled water was used as the baseline to complete hemolysis, while Trolox was used as the positive control and PBS as the negative control. As was done by Takebayashi et al. (2012) [54], 200 µL of sheep erythrocytes in 2.8% PBS were added and the plate was incubated at 37 ± 0.5 °C for 10 min with shaking; then, 200 µL of a solution of 2,2-azobis(2-amidinopropane) dihydrochloride (AAPH 160 mM in PBS) and initial and 1 h-post readings of the optical density were taken on an ELX800 microplate reader (Bio-Tek Instruments, Winooski, VT, USA) at 690 nm. Finally, it was incubated again, performing readings every 10 min for approximately 300 min and maintaining the mentioned conditions. The calculation of erythrocyte population that remained intact (P) was performed using Equation (2), where S_0_ and S_t_ correspond to the optical densities of the samples at 0 and t minutes, respectively, and CH_0_ corresponds to the optical density of complete hemolysis at time 0.

P(%) = (S_t_ − CH_0_)/(S_0_ − CH_0_) × 100 (2)

The results are expressed in time required to produce hemolysis (Δt), calculated by Equation (3), where Ht_50_ is the hemolytic time of 50% of the erythrocyte population, obtained from the hemolysis curve plotted for each dilution of the extract.

Δt = Ht_50(sample)_ − Ht_50(control)_ (3)

The Δt values were correlated with the different concentrations of the extracts, determining the concentration of extract required to retard hemolysis at a fixed time. Likewise, the concentration required to delay hemolysis (IC_50_, μg/mL) at 60 and 120 min was calculated.

#### 3.4.3. Diphenyl-1-picrylhydrazyl Radicals Scavenging (DPPH)

As described by Brand-Williams et al. (1995) [55], a solution of 2,2-diphenyl-1-picrylhydrazyl (DPPH) was prepared at a concentration of 6.0 × 10^−5^ M in methanol. In 96-well microplates, 30 µL of extract dissolved in distilled water was added and serial dilution was performed in order to obtain 8 distinct concentrations, depending on the extract tested and in triplicate. The extraction solvent was used as the negative control, with Trolox being used as the positive one. After that, 270 µL of the DPPH solution was added in each well and kept for 1 h at room temperature and in the dark. Afterwards, the plate was taken for absorbance reading in spectrophotometer (SPECTROstar Nano, BMG LABTECH, Ortenberg, Germany) at 515 nm. The calculation of the IC_50_ value was undertaken similarly to the method applied for the TBARS method.

### 3.5. Antimicrobial Activity—Microdilution Method

The antimicrobial potential of the extracts was evaluated following the methodology outlined by Rotilie et al. (1975) [56] for the following set of microorganisms acquired from the company Frilabo in Porto, Portugal: Gram-negative bacteria—*Enterobacter cloacae* (ATCC 49741), *Escherichia coli* (ATCC 25922), *Pseudomonas aeruginosa* (ATCC 9027), *Salmonella enterica subsp* (ATCC 13076), *Yersinia enterocolitica* (ATCC 8610); Gram-positive bacteria—*Bacillus cereus* (ATCC 11778), *Listeria monocytogenes* (ATCC 19111), *Staphylococcus aureus* (ATCC 204305) and fungi—*Aspergillus fumigatus* (ATCC 204305) and *Aspergillus brasiliensis* (ATCC 16404). The microorganisms were previously incubated under different conditions in order to obtain them in their exponential growth phase. The bacteria *E. coli*, *S. enterica*, *P. aeruginosa* and *Y. enterocolitica* were incubated at 37 ± 0.5 °C in MacConkey agar culture medium for 24 h. The other bacteria were incubated under the same conditions, but in blood agar medium. The fungi were incubated in Malt Extract Broth (MEB) medium at 25 ± 0.5 °C for 72 h. Suspensions of the bacteria were prepared on Tryptic Soy Broth (TSB) medium standardized at 1.5 × 10^6^ CFU/mL and quantified using a densitometer DEN-1B (Biosan, Riga, Latvia). Suspensions of the fungi were prepared in PBS and TWEEN (0.1%) standardized at 1.0 × 10^6^ CFU/mL, using a Neubauer chamber. A stock solution of 20 mg/mL of extract was prepared in DMSO (5%; *v*/*v*) and TSB culture medium. In a 96-well microplate, 90 μL of the extract solution was added into 100 μL of TSB and serial dilutions were performed. Subsequently, 10 μL of inoculum was added in each of the wells, obtaining effectively tested extract concentrations, in duplicates, between 10 and 0.075 mg/mL. Negative controls of TSB culture medium and of the extract in TSB were prepared. Streptomycin, methicillin (1–0.007 mg/mL) and ampicillin (10–0.07 mg/mL) antibiotics for bacteria and ketoconazole (1–0.007 mg/mL) for fungi were used as positive controls. The plates with bacteria were covered and incubated at 37 ± 0.5 °C for 24 h. After this period, 40 μL of a 0.2 mg/mL solution of the colorimetric indicator *p*-iodonitrotetrazolium chloride (INT) prepared in sterile water was added and the plate was incubated at 37 ± 0.5 °C for 30 min. The minimum inhibitory concentration (MIC) was defined as the lowest concentration that inhibits visible bacterial growth determined by the change in coloration from yellow to pink if the microorganisms are viable. For the determination of minimum bactericidal concentration (MBC), defined as the lowest concentration required to kill the bacteria, 50 μL of liquid from each well that showed no color change was seeded onto solid medium and incubated at 37 ± 0.5 °C for 24 h. The lowest concentration that produced no growth determined the MBC. The plate with fungi was incubated at 25 ± 0.5 °C for 72 h. After this period, the MIC was determined directly via comparison with the positive control to identify the lowest concentration in which there was no visible fungal growth, determined by the visualization of spores. To determine the minimum fungicidal concentration (MFC), defined as the lowest concentration needed to kill a fungus, the plate was incubated for another 72 h at 25 ± 0.5 °C and a new observation was made to check for visible fungal growth.

### 3.6. Cytotoxicity in Tumor and Non-Tumor Cell Lines

The cytotoxicity of the extracts was evaluated according to the methodology used by Mandim et al. (2018) [32]. The tumor cell lines AGS (gastric adenocarcinoma), CaCo-2 (colorectal adenocarcinoma) and MCF-7 (breast adenocarcinoma), and the non-tumor cell lines VERO (African green monkey kidney) and PLP_2_ (pig liver primary culture), were used. All cell lines were obtained from the Leibniz Institute DSMZ—German Collection of Microorganisms and Cell Cultures, except PLP_2_, which was obtained from the liver of a pig slaughtered in a local abattoir. The maintenance of the VERO line was performed in DMEM medium, and the maintenance of the other cell lines was performed in RPMI-1640 medium, both supplemented with fetal bovine serum (10%), glutamine (2 mM), and the antibiotics penicillin (100 U/mL) and streptomycin (100 mg/mL). The culture flasks were incubated at 37 ± 0.5 °C in humid atmosphere with 5% CO_2_, and cells were used when they presented between 70 and 80% confluence. A stock solution of extract in sterile water of 8 mg/mL was prepared from which serial dilutions were made, obtaining concentrations between 8 and 0.125 mg/mL. In 96-well microplates, 10 μL of each of the extract concentrations and 190 μL of the cell line suspension were added in duplicates, the final concentrations tested being between 400 and 6.25 µg/mL. Ellipticine was used as the positive control and the cell suspension without the addition of other components was used as a negative control. After checking the adherence of the cells, the microplates were incubated for a period of 72 h at 37 ± 0.5 °C in a humid environment with 5% CO_2_. The VERO line was tested at a density of 1.9 × 10^4^ cells/well and the other cell lines at a density of 1.0 × 10^4^ cells/well. After incubation, the reaction was stopped with 100 μL of previously cooled trichloroacetic acid (10%; *w*/*v*). The plates were incubated at 4 ± 0.5 °C for 1 h, washed with water, and after drying, 100 μL of SRB (0.057%; *w*/*v*) was added and left for 30 min at room temperature. Non-adhered SRB was removed by washing with acetic acid solution (1%; *v*/*v*), and adhered SRB was solubilized with 200 μL of Tris (10 mM). The absorbance values were read from the spectrophotometer (ELX800 microplate reader, Bio-Tek Instruments, Winooski, VT, USA) at 540 nm. The results are expressed in terms of the concentration of extract able to inhibit cell proliferation by 50% (GI_50_).

### 3.7. Artemia Franciscana Acute Toxicity Test

*Artemia franciscana* cysts were obtained from the ArtoxKit M (MicroBio Tests, Gent, Belgium). Using 60 μm mesh, the cysts were washed with abundant tap water and were hydrated for about 2 h in distilled water until a rounded shape was observed. The hydrated cysts were incubated in medium simulating seawater, prepared according to the methods of Sorgeloos et al. (1996) [57], under constant aeration and light (3000–4000 lux) at a temperature of 21.5 ± 0.5 °C until hatching. Instar II-III nauplii were used in the assay. Solutions of the plant extract in incubation medium were prepared at concentrations of 400 and 200 mg/mL, in line with concentrations previously tested in a cytotoxicity assay. A stock solution of potassium dichromate (K_2_Cr_2_O_7_; 1000 mg/L) was used as reference toxicant for preparing different concentrations in an incubation medium (100, 56, 32, 18, and 10 mg/L) for the assay sensitivity control (negative control). The positive control was the incubation medium. The plant extract solutions and controls were aerated for 2 h before starting the assay on the plates. Test plate wells were filled with plant extract solution, with 4 replicates for each concentration tested and with the controls. Nauplii were collected and then transferred to the test wells, with 5 or 6 individuals added in each, ensuring an observation group of at least 20 individuals per concentration tested. The plates were kept in the dark, at a temperature of 21.5 ± 0.5 °C, and the number of immobilized individuals was counted at 12 and 24 h of the assay. The results are expressed in relation to the range in which the concentration is lethal to 50% of the test organism population (LC_50_), based on mortality at the end of 24 h.

### 3.8. Statistical Analysis

The variable *n* was used to denote the number of replicates for each assay, described in the legends of the tables. The results are expressed as mean value ± standard deviation (SD) and the statistical treatment was performed by analysis of variance (ANOVA) followed by Tukey’s HSD and Fischer LSD tests using the software RStudio version 2023.06.1. The calculation for the LC_50_ parameter, applicable to the negative control of the acute toxicity test with *Artemia franciscana* (potassium dichromate), was performed considering the data on the percentage of deaths *per* concentration tested, at 24 h, through the linear regression of the Log_10_ of the concentration as a function of the Probit values.

## 4. Conclusions

Our investigation focused on identifying natural substitutes for chemical additives in the food and cosmetic industries, specifically focusing on extracts with potent antioxidant and antimicrobial properties. In this context, the performance of the HIR extract in TBARS (IC_50_ = 1.2 ± 0.1 µg/mL) and DPPH (IC_50_ = 5.3 ± 0.4 µg/mL) assays stands out. HIR was found to show inhibitory activity for all tested bacteria and fungi, especially against *L. monocytogenes* and *S. aureus* (MIC = 0.6 mg/mL). This reveals the high potential applicability of this extract for contamination control. 

The safe replacement of chemical additives by components of natural origin in products requires knowledge on the toxicity profiles of the extracts. Considering the cytotoxicity assay with liver and kidney cell lines, JUR showed some toxic effect on renal cells (GI_50_ = 69 ± 1 µg/mL; VERO), while the results suggest greater harmlessness for VIV and SAN, which agrees with what was observed in the assay with *A. franciscana* (LC_50_ > 400 mg/L). The results obtained for the in vivo assay suggest that the extracts CAV and CAO present LC_50_ values within the range of concentrations tested (200–400 mg/L), it being necessary to perform the assays with intermediate concentrations to determine the exact value for LC_50_. For the other plants extract, it was necessary to test at higher concentrations to determine this parameter.

As regards opportunities for innovation in industrial applications, HIR extract appears to be the most promising due to its high antioxidant capacity, good overall performance in inhibiting the tested contaminating bacteria, low toxicity, and high content of phenolic compounds.

## Figures and Tables

**Table 1:** Identification and quantification of phenolic compounds (*n* = 2) from the extracts of the studied plant species. The retention time (Rt), wavelengths of maximum absorption in the visible region (λmax) and mass spectral data are presented. Most quantified compound highlighted in bold. Results are expressed in mg/g dry extract, mean ± standard deviation.

Peak	Rt (min)	λmax (nm)	[M-H]^−^ (*m*/*z*)	Tentative Identification	Content (mg/g)
*Calendula officinalis* L.					
**1** *^G^*	6.31	322	353	**5-*O*-Caffeoylquinic acid**	8.4 ± 0.5
**2** *^A^*	8.71	326	341	Caffeic acid hexoside	0.61 ± 0.01
**3** *^F^*	13.5	346	755	Quercetin-3-*O*-rhamnosylrutinoside	0.737 ± 0.001
**4** *^F^*	14.99	350	609	Quercetin-3-*O*-rutinoside	1.01 ± 0.02
**5** *^J^*	15.93	354	769	Isorhamnetin-3-*O*-rhamnosylrutinoside	0.65 ± 0.04
**6** *^F^*	16.69	353	595	Quercetin-*O*-pentosylhexoside	1.26 ± 0.02
**7** *^F^*	16.67	353	623	Isorhamnetin-3-*O*-neohesperidoside	1.31 ± 0.02
**8** *^F^*	19.17	346	549	Quercetin-7-*O*-malonylhexoside	0.99 ± 0.03
**9** *^F^*	19.84	343	593	Kaempferol-*O*-rutinoside	0.660 ± 0.002
**10** *^F^*	20.82	354	623	Isorhamnetin-3-*O*-rutinoside	3.5 ± 0.2
**11** *^F^*	22.02	343	477	Isorhamnetin-3-*O*-glucoside	0.86 ± 0.01
**12** *^F^*	24.64	353	519	Isorhamnetin-3-*O*-(6″-acetyl)-glucoside	1.06 ± 0.03
				**Total Phenolic Acids**	9 ± 0.5
				**Total Flavonols**	12.1 ± 0.4
				**Total Phenolic Compounds**	21.0 ± 0.8
*Calluna vulgaris* (L.) Hull					
**1** *^G^*	4.36	324	353	3-*O*-Caffeoylquinic acid	4.70 ± 0.03
**2** *^L^*	4.94	279	575	Proanthocyanidin dimer A type	5.56 ± 0.08
**3** *^L^*	5.32	287	1151	Procyanidin tetramer A type	7.67 ± 0.04
**4** *^G^*	6.34	324	707	**Dimer of 3-*O*-Caffeoylquinic acid**	27.5 ± 0.5
**5** *^G^*	7.41	321	353	5-*O*-Caffeoylquinic acid	4.90 ± 0.01
**6** *^F^*	8.64	340	465	Dihydroquercetin-6-*C*-hesoxide	1.72 ± 0.03
**7** *^F^*	9.77	341	465	Dihydroquercetin-*C*-hesoxide	0.83 ± 0.02
**8** *^O^*	13.08	285	435	Taxifolin-*O*-pentoside	4.026 ± 0.003
**9** *^L^*	14.35	283	1153	Procyanidin dimer B-type	6.8 ± 0.1
**10** *^F^*	14.73	350	595	Quercetin-*O*-pentosyl-hexoside	1.50 ± 0.02
**11** *^F^*	16.49	350	493	Myricetin-3-*O*-glucuronide	1.43 ± 0.03
**12** *^F^*	17.36	355	463	Quercetin-3-*O*-glucoside	6.11 ± 0.06
**13** *^F^*	19.26	350	609	Quercetin-3-*O*-rutinoside	1.18 ± 0.02
**14** *^F^*	20.14	352	433	Quercetin-*O*-pentoside	2.11 ± 0.02
**15** *^F^*	21.1	347	477	Quercetin-3-*O*-glucuronide	1.21 ± 0.02
**16** *^F^*	21.9	341	447	Quercetin-*O*-rhamnoside	1.00 ± 0.01
**17** *^M^*	23.15	346	621	Luteolin acetyl pentosyl-hexoside	2.1 ± 0.1
				**Total Phenolic Acids**	37.1 ± 0.5
				**Total Flavan-3-ols**	20.1 ± 0.2
				**Total Flavones**	2.1 ± 0.1
				**Total Flavonols**	17.1 ± 0.2
				**Total Flavononols**	4.026 ± 0.003
				**Total Phenolic Compounds**	80 ± 1
*Hippophae rhamnoides* L.					
**1** *^I^*	5.61	274	633	**Galloyl-HHDP-glucose**	56.3 ± 0.9
**2** *^I^*	6.31	276	935	Galloyl-bis-HHDP-glucose isomer I	0.61 ± 0.03
**3** *^P^*	7.73	275	577	Procyanidin dimer	51 ± 1
**4** *^L^*	9.12	274	865	Procyanidin trimer	1.10 ± 0.01
**5** *^F^*	10.62	350	831	Isorhamnetin-*O*-hydroxyferuloylhexoside-*O*-hexoside	1.32 ± 0.02
**6** *^L^*	11.48	274	1441	B-type (*epi*)catechin pentamer	0.98 ± 0.03
**7** *^I^*	11.89	275	935	Galloyl-bis-HHDP-glucose	0.184 ± 0.007
**8** *^F^*	12.56	352	463	Quercetin-3-*O*-glucoside	0.96 ± 0.03
**9** *^L^*	13.51	275	865	Procyanidin trimer	0.887 ± 0.001
**10** *^L^*	14.58	281	1153	Procyanidin tetramer	1.31 ± 0.05
**11** *^I^*	15.02	275	1567	Saguiin H10	0.47 ± 0.02
**12** *^F^*	16.63	350	609	Quercetin-3-*O*-rutinoside	1.37 ± 0.03
**13** *^H^*	17.31	362	433	Ellagic acid pentoside	1.70 ± 0.03
**14** *^F^*	17.67	282	935	Quercetin-*O*-glucosyl-glucoside	1.60 ± 0.02
**15** *^I^*	18.29	350	961	Galloyl-bis-HHDP-glucose isomer II	1.12 ± 0.04
**16** *^F^*	20.82	353	623	Isorhamnetin-3-*O*-rutinoside	1.24 ± 0.03
**17** *^F^*	22.01	350	477	Isorhamnetin-3-*O*-glucoside	0.87 ± 0.02
**18** *^F^*	31.9	338	593	Kaempferol-*O*-rutinoside	0.581 ± 0.003
				**Total Phenolic Acids**	1.70 ± 0.03
				**Total Flavonols**	7.9 ± 0.2
				**Total Ellagitannins**	59 ± 1
				**Total Flavan-3-ols**	55 ± 1
				**Total Phenolic Compounds**	123 ± 2
*Juglans regia* L.					
**1** *^G^*	4.28	322	353	**3-*O*-Caffeoylquinic acid**	25.0 ± 0.4
**2** *^G^*	5.57	312	337	*cis* 4*-p*-Coumaroylquinic acid	12.4 ± 0.4
**3** *^G^*	6.28	324	353	5-*O*-Caffeoylquinic acid	11.8 ± 0.5
**4** *^G^*	7.32	311	337	*trans* 4-*p*-Coumaroylquinic acid	3.99 ± 0.03
**5** *^G^*	8.57	314	337	*cis* 5-*p*-Coumaroylquinic acid	3.89 ± 0.02
**6** *^G^*	10.54	325	337	*trans* 5-*p*-Coumaroylquinic acid	5.20 ± 0.03
**7** *^F^*	16.51	331	463	Quercetin-3-*O*-glucoside	0.92 ± 0.01
**8** *^F^*	17.31	354	463	Quercetin-3-*O*-hexoside	13.4 ± 0.1
**9** *^F^*	19.17	350	433	Quercetin-*O*-pentoside	1.82 ± 0.02
**10** *^F^*	20.01	351	433	Quercetin-*O*-pentoside	7.11 ± 0.08
**11** *^F^*	21.08	348	447	Quercetin-3-*O*-rhamnoside	3.5 ± 0.2
**12** *^C^*	22.85	344	417	Luteolin-*O*-pentoside	4.0 ± 0.1
**13** *^A^*	24.29	328	501	Caffeic acid derivative	0.68 ± 0.02
**14** *^F^*	25.69	332	431	Kaempferol-*O*-deoxyhexosyl	0.89 ± 0.01
**15** *^F^*	26.71	340	489	Acetylquercetin-*O*-rhamnoside isomer I	0.90 ± 0.01
**16** *^F^*	28.67	341	489	Acetylquercetin-*O*-rhamnoside isomer II	0.92 ± 0.03
				**Total Phenolic Acids**	67 ± 1
				**Total Flavonols**	29.5 ± 0.5
				**Total Phenolic Compounds**	96 ± 2
*Mentha cervina* L.					
**1** *^D^*	4.16	278	197	Syringic acid	0.89 ± 0.01
**2** *^L^*	6.51	318	305	Gallocatechin	2.42 ± 0.07
**3** *^B^*	8.23	322	313	Salvianolic acid F	1.48 ± 0.03
**4** *^E^*	8.79	319	593	Apigenin 6,8-*C*-diglucoside	5.18 ± 0.07
**5** *^B^*	13.35	322	537	Lithospermic acid A isomer I	2.61 ± 0.05
**6** *^B^*	14.52	320	539	Yannaneic acid D isomer I	4.32 ± 0.04
**7** *^B^*	16.25	321	539	Yannaneic acid D isomer II	4.7 ± 0.3
**8** *^B^*	17.41	321	717	Salvianolic acid A	6.0 ± 0.3
**9** *^B^*	19.98	321	719	Sagerinic acid	2.68 ± 0.04
**10** *^B^*	20.48	327	717	**Salvianolic acid L**	28.7 ± 0.3
**11** *^B^*	21.87	328	359	*cis*-Rosmarinic acid	5.37 ± 0.03
**12** *^B^*	22.87	331	359	*trans*-Rosmarinic acid	2.54 ± 0.02
**13** *^B^*	23.95	325	537	Lithospermic acid A isomer II	3.28 ± 0.04
**14** *^B^*	25.89	322	521	Rosmarinic acid hexoside	1.33 ± 0.03
**15** *^B^*	29.73	323	537	Lithospermic acid A isomer III	6.11 ± 0.08
				**Total Phenolic Acids**	70 ± 1
				**Total Flavan-3-ols**	2.42 ± 0.07
				**Total Flavones**	5.18 ± 0.07
				**Total Phenolic Compounds**	78 ± 1
*Rubus idaeus* L.					
**1** *^G^*	4.34	324	353	3-*O*-Caffeoylquinic acid	7.8 ± 0.1
**2** *^K^*	5.15	323	417	Dihydroxybenzoic acid-*O*-dipentoside	6.9 ± 0.4
**3** *^A^*	5.66	321	341	Caffeic acid hexoside	3.4 ± 0.1
**4** *^L^*	8.28	280	577	Procyanidin dimer	10.13 ± 0.05
**5** *^C^*	9.62	322	401	Apigenin-*O*-pentoside	1.337 ± 0.001
**6** *^H^*	12.21	280	1401	Lambertianin C	11.7 ± 0.2
**7** *^I^*	12.82	280	935	**Galloyl-bis-HHDP-glucoside**	36.8 ± 0.4
**8** *^H^*	16.54	364	433	Ellagic acid pentoside	1.78 ± 0.02
**9** *^F^*	17.21	352	477	Quercetin-*O*-glucuronide	4.5 ± 0.2
**10** *^F^*	19.01	324	607	Kaempferol glucuronyl-rhamnoside	1.11 ± 0.03
**11** *^F^*	19.84	325	593	Kaempferol-*O*-rutinoside	0.97 ± 0.02
**12** *^M^*	20.74	342	461	Luteolin-glucuronide	11.65 ± 0.05
**13** *^F^*	22.47	330	461	Kaempferol-*O*-glucoronide	1.24 ± 0.03
**14** *^F^*	22.82	326	447	Quercetin-3-*O*-rhamnoside	0.95 ± 0.02
				**Total Phenolic Acids**	19.9 ± 0.7
				**Total Flavonols**	8.8 ± 0.3
				**Total Flavones**	12.99 ± 0.05
				**Total Flavan-3ols**	10.13 ± 0.05
				**Total Ellagitannins**	48.6 ± 0.6
				**Total Phenolic Compounds**	100 ± 2
*Sambucus nigra* L.					
**1** *^G^*	4.17	322	353	3-*O*-Caffeoylquinic acid	5.01 ± 0.03
**2** *^G^*	6.18	336	353	**5-*O*-Caffeoylquinic acid**	52.0 ± 0.3
**3** *^A^*	8.76	323	179	Caffeic acid	1.19 ± 0.03
**4** *^N^*	10.09	312	337	*p*-Coumaroylquinic acid	2.17 ± 0.03
**5** *^Q^*	12.23	318	367	Feruloyl-quinic acid	0.45 ± 0.01
**6** *^F^*	13.01	331	625	Quercetin-diglucoside	0.680 ± 0.003
**7** *^F^*	13.97	325	755	Kaempferol-*O*-hexosyl-*O*-rutinoside	0.554 ± 0.003
**8** *^F^*	15.69	324	639	Isorhamnetin dihexoside	0.72 ± 0.03
**9** *^F^*	16.53	356	609	Quercetin-3-*O*-rutinoside	24.9 ± 0.4
**10** *^F^*	17.74	353	609	Quercetin-deoxyhexosylhexoside	3.9 ± 0.2
**11** *^F^*	18.94	343	549	Quercetin-7-*O*-malonylhexoside	3.8 ± 0.2
**12** *^F^*	19.85	341	593	Kaempferol-*O*-rutinoside	2.00 ± 0.02
**13** *^F^*	20.88	354	623	Isorhamnetin-3-*O*-rutinoside	5.5 ± 0.3
**14** *^F^*	21.94	346	477	Kaempferol-3-*O*-glucoside	2.495 ± 0.008
**15** *^F^*	24.59	349	519	Isorhamnetin-3-*O*-acetyl-glucoside	1.08 ± 0.02
				**Total Phenolic Acids**	60.8 ± 0.4
				**Total Flavonols**	46 ± 1
				**Total Phenolic Compounds**	106 ± 2
*Vitis vinifera* L.					
**1** *^A^*	4.55	328	311	**Caftaric acid**	4.03 ± 0.02
**2** *^A^*	5.92	311	295	*Cis*-Coutaric acid	1.522 ± 0.009
**3** *^A^*	6.53	315	295	*Trans*-Coutaric acid	1.52 ± 0.01
**4** *^F^*	17.28	354	477	Quercetin-glucoronide	3.29 ± 0.02
**5** *^A^*	17.73	354	463	Quercetin-3-*O*-glucoside	3.72 ± 0.09
**6** *^F^*	19.91	346	593	Kaempferol-*O*-rutinoside	0.89 ± 0.02
**7** *^F^*	21.07	346	447	Kaempferol-7-*O*-hexoside	1.062 ± 0.006
				**Total Phenolic Acids**	7.1 ± 0.1
				**Total Flavonols**	8.97 ± 0.05
				**Total Phenolic Compounds**	16.0 ± 0.2

Standards used in quantification: *^A^* caffeic acid; *^B^* rosmarinic acid; *^C^* apigenin-7-*O*-glucoside; *^D^* syringic acid; *^E^* apigenin-6-*C*-glucoside; *^F^* quercetin-3-*O*-glucoside; *^G^* chlorogenic acid; *^H^* ellagic acid; *^I^* gallic acid; *^J^* quercetin-3-*O*-rutinoside; *^K^* hydroxybenzoic acid; *^L^* catechin; *^M^* luteolin-6-*C*-glucoside; *^N^ p*-coumaric acid; *^O^* taxifolin; *^P^* (*epi*)catechin; *^Q^* ferulic acid.

**Table 2:** Results of antioxidant activity (*n* = 3) for the evaluated extracts. Best performances highlighted in bold.

	CAO	CAV	HIR	JUR	MEC	RUI	SAN	VIV
	Antioxidant Activity—IC_50_ (µg/mL)							
TBARS	129 ± 22 ^a^	6 ± 1 ^d^	**1.2 ± 0.1** ^d^	25 ± 1 ^c^	50 ± 3 ^b^	6 ± 0.3 ^d^	123 ± 4 ^a^	9 ± 1 ^d^
DPPH	64 ± 3 ^a^	8.7 ± 0.3 ^f^	**5.3 ± 0.4** ^g^	26.39 ± 0.04 ^c^	32 ± 1 ^b^	9.9 ± 0.3 ^f^	14.0 ± 0.3 ^d^	11.7 ± 0.2 ^e^
OxHLIA (Δt = 60 min)	266 ± 12 ^a^	23 ± 1 ^e^	43 ± 1 ^d^	27 ± 1 ^e^	112 ± 6 ^b^	88 ± 3 ^c^	28 ± 1 ^e^	**22 ± 1** ^e^
OxHLIA (Δt = 120 min)	500 ± 14 ^a^	47 ± 1 ^e^	79 ± 1 ^d^	51 ± 1 ^e^	331 ± 14 ^b^	222 ± 4 ^c^	**44 ± 1** ^e^	49 ± 1 ^e^

CAO, *C. officinalis*; CAV, *C. vulgaris*; HIR, *H. rhamnoides*; JUR, *J. regia*; MEC, *M. cervina*; RUI, *R. idaeus*; SAN, *S. nigra*; VIV, *V. vinifera*. IC_50_ values for Trolox (µg/mL): 23 ± 0.4 (TBARS); 42 ± 1 (DPPH); 22 ± 1 (OxHLIA Δt = 60 min); 44 ± 1 (OxHLIA Δt = 120 min). Different letters represent significant differences (*p* < 0.05).

**Table 3:** Results of antimicrobial activity (*n* = 2) for the evaluated extracts. Best performances highlighted in bold.

	CAO	CAV	HIR	JUR	MEC	RUI	SAN	VIV
	Antimicrobial Activity—MIC (mg/mL)							
Gram-negative Bacteria								
*E. cloacae*	>10	5	**2.5**	10	>10	**2.5**	10	10
*E. coli*	>10	>10	**10**	>10	>10	**10**	>10	>10
*P. aeruginosa*	>10	10	**5**	10	>10	10	>10	10
*S. enterica*	10	10	**2.5**	10	10	**2.5**	10	10
*Y. enterocolitica*	10	10	**1.25**	2.5	10	5	10	5
Gram-positive Bacteria								
*B. cereus*	2.5	10	**1.25**	5	10	5	5	10
*L. monocytogenes*	2.5	2.5	**0.6**	2.5	10	2.5	1.25	10
*S. aureus*	5	5	**0.6**	2.5	10	2.5	10	5
Fungi								
*A. brasiliensis*	10	10	10	**1.25**	5	5	10	10
*A. fumigatus*	10	10	10	10	10	10	10	10

CAO, *C. officinalis*; CAV, *C. vulgaris*; HIR, *H. rhamnoides*; JUR, *J. regia*; MEC, *M. cervina*; RUI, *R. idaeus*; SAN, *S. nigra*; VIV, *V. vinifera*. MIC, MBC and MFC values for the positive controls (mg/mL): streptomycin (0.007–0.06); methicillin (0.007); ampicillin (0.15–0.63); ketoconazole (0.06–1).

**Table 4:** Cytotoxicity (*n* = 2) in tumoral cell lines (AGS, CaCo-2, MCF-7) and non-tumoral cell lines (VERO, PLP_2_) and acute toxicity in *A. franciscana* (20 ≤ *n* ≤ 23) for the plant extracts under study.

	Cytotoxicity—GI_50_ (µg/mL)					Acute Toxicity *A. franciscana* LC_50_ (mg/L)
	Tumoral			Non-Tumoral		
	AGS	CaCo-2	MCF-7	VERO	PLP_2_	
CAO	340 ± 17 ^a^	213 ± 20 ^bc^	241 ± 17 ^ab^	261 ± 27 ^b^	214 ± 7 ^d^	200 < LC_50_ < 400
CAV	124 ± 2 ^e^	282 ± 21 ^a^	274 ± 19 ^a^	257 ± 24 ^b^	261 ± 10 ^b^	200 < LC_50_ < 400
HIR	183 ± 11 ^d^	194 ± 11 ^bc^	209 ± 11 ^b^	219 ± 19 ^b^	178 ± 3 ^e^	>400
JUR	231 ± 21 ^c^	219 ± 4 ^b^	253 ± 12 ^a^	69 ± 1 ^c^	193 ± 18 ^de^	>400
MEC	277 ± 25 ^b^	297 ± 21 ^a^	>400	228 ± 8 ^b^	238 ± 10 ^c^	>400
RUI	134 ± 7 ^e^	174 ± 17 ^c^	208 ± 16 ^b^	>400	175 ± 6 ^e^	>400
SAN	>400	>400	>400	314 ± 5 ^a^	381 ± 3 ^a^	>400
VIV	313 ± 30 ^ab^	>400	>400	>400	>400	>400

CAO, *C. officinalis*; CAV, *C. vulgaris*; HIR, *H. rhamnoides*; JUR, *J. regia*; MEC, *M. cervina*; RUI, *R. idaeus*; SAN, *S. nigra*; VIV, *V. vinifera*. GI_50_ values for ellipticine (µg/mL): gastric adenocarcinoma (AGS)—1.23 ± 0.03; colorectal adenocarcinoma (CaCo-2)—1.21 ± 0.02; breast adenocarcinoma (MCF-7)—1.02 ± 0.02; African green monkey kidney (VERO)—1.4 ± 0.1; pig liver primary culture (PLP_2_)—1.41 ± 0.06. LC_50_ for potassium dichromate: 48 ± 4 mg/L. Different letters represent significant differences (*p* < 0.05).

## Data Availability

The data presented in this study are available in this article.
